# A Case of an Acutely Ill Adult Athlete with Previously Undiagnosed Hypertrophic Obstructive Cardiomyopathy

**DOI:** 10.7759/cureus.4875

**Published:** 2019-06-10

**Authors:** Michael R Minckler, Madeleine Maker

**Affiliations:** 1 Emergency Medicine, Providence St. Mary Medical Center, Walla Walla, USA; 2 Miscellaneous, Whitman College, Walla Walla, USA

**Keywords:** hypertrophic obstructive cardiomyopathy, lvot, athlete, athletics, shortness of breath

## Abstract

Hypertrophic obstructive cardiomyopathy (HOCM) is a genetic condition most commonly characterized by hypertrophy of the ventricular septum, which leads to left ventricle outflow obstruction. Due to the severity of the condition, it is often diagnosed in adolescents, especially in those who exercise. We describe the case of a 53-year-old male, previously undiagnosed with hypertrophic cardiomyopathy (HCM), who became dyspneic during a bike race. He was found to have elevated troponin, pulmonary edema, and was diagnosed with HOCM. The late presentation of the case, in an active individual, makes the situation unique.

## Introduction

Hypertrophic cardiomyopathy (HCM) is a relatively common genetic condition. It is a result of one or more missense mutations in genes encoding proteins of the cardiac sarcomere [1]. The condition affects 1 in 500 in the general population, disproportionately affecting athletes [2-3]. It is most commonly characterized by hypertrophy of the ventricular septum, though there are other less common types [4]. In 20% to 25% of patients, left ventricular (LV) outflow obstruction may occur, which is caused by impingement of the mitral valve leaflets on the hypertrophied basal septum and a pressure gradient that pulls the mitral valve anteriorly [3,5]. Depending on the severity of the condition, patients may be asymptomatic, though end-stage patients may present with symptoms mirroring a heart attack, systolic dysfunction, ventricular arrhythmias, valvular stenosis, and sudden death [2,6]. Due to the severity of the condition, it most commonly becomes apparent during puberty, when growth spurts occur [1].

## Case presentation

Our patient was a 53-year-old man participating in a day-long bike race. His past medical history was limited to hypertension, and controlled with amlodipine. During the race, he was severely short of breath and presented to the medical tent. He noted some subjective improvement with a single dose of 2.5 mg albuterol but his shortness of breath persisted triggering his visit to a critical access emergency room (ER). He did not experience any associated pain.

**Figure 1:**
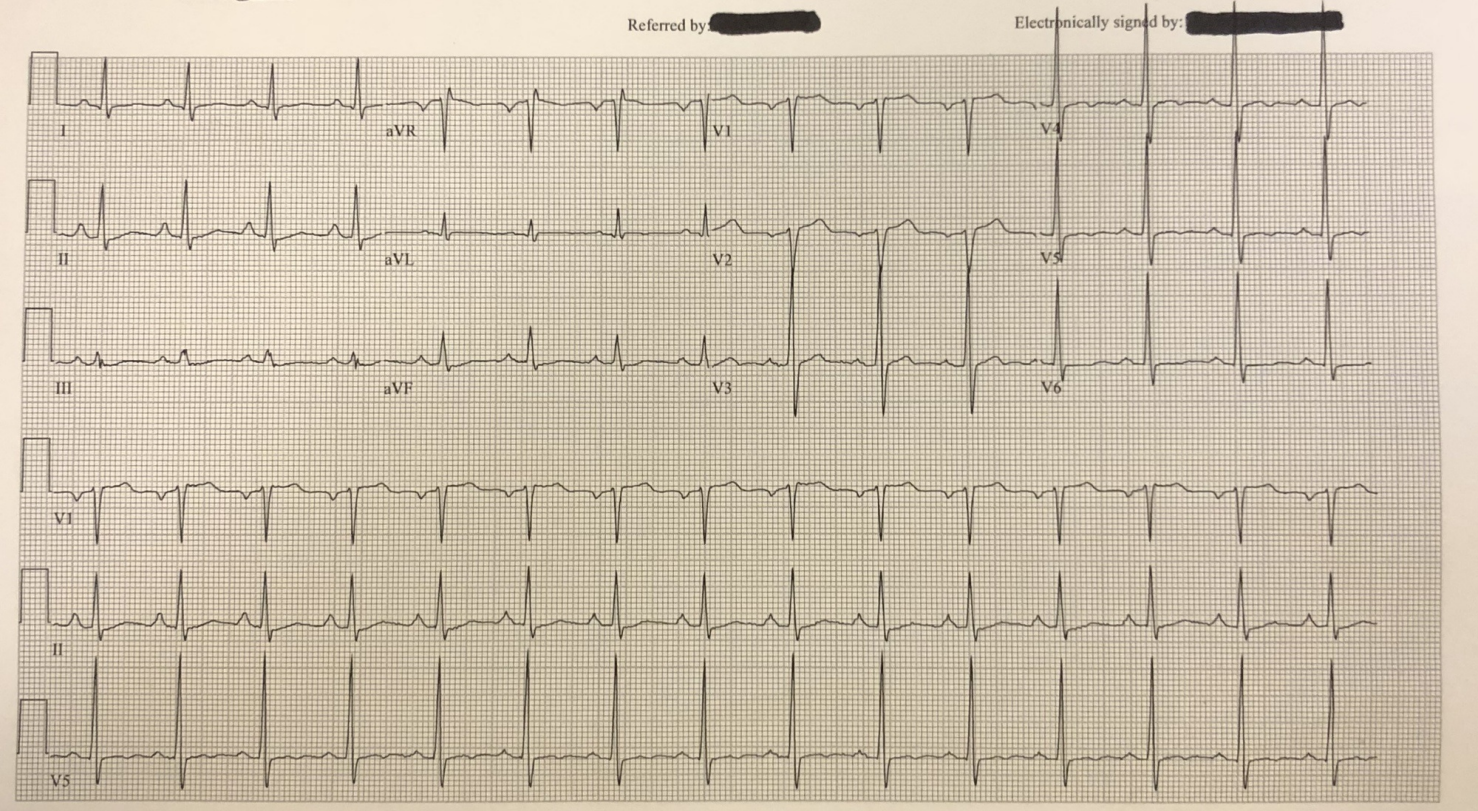
Electrocardiogram (EKG) displaying hyperdynamic QRS waves

**Figure 2:**
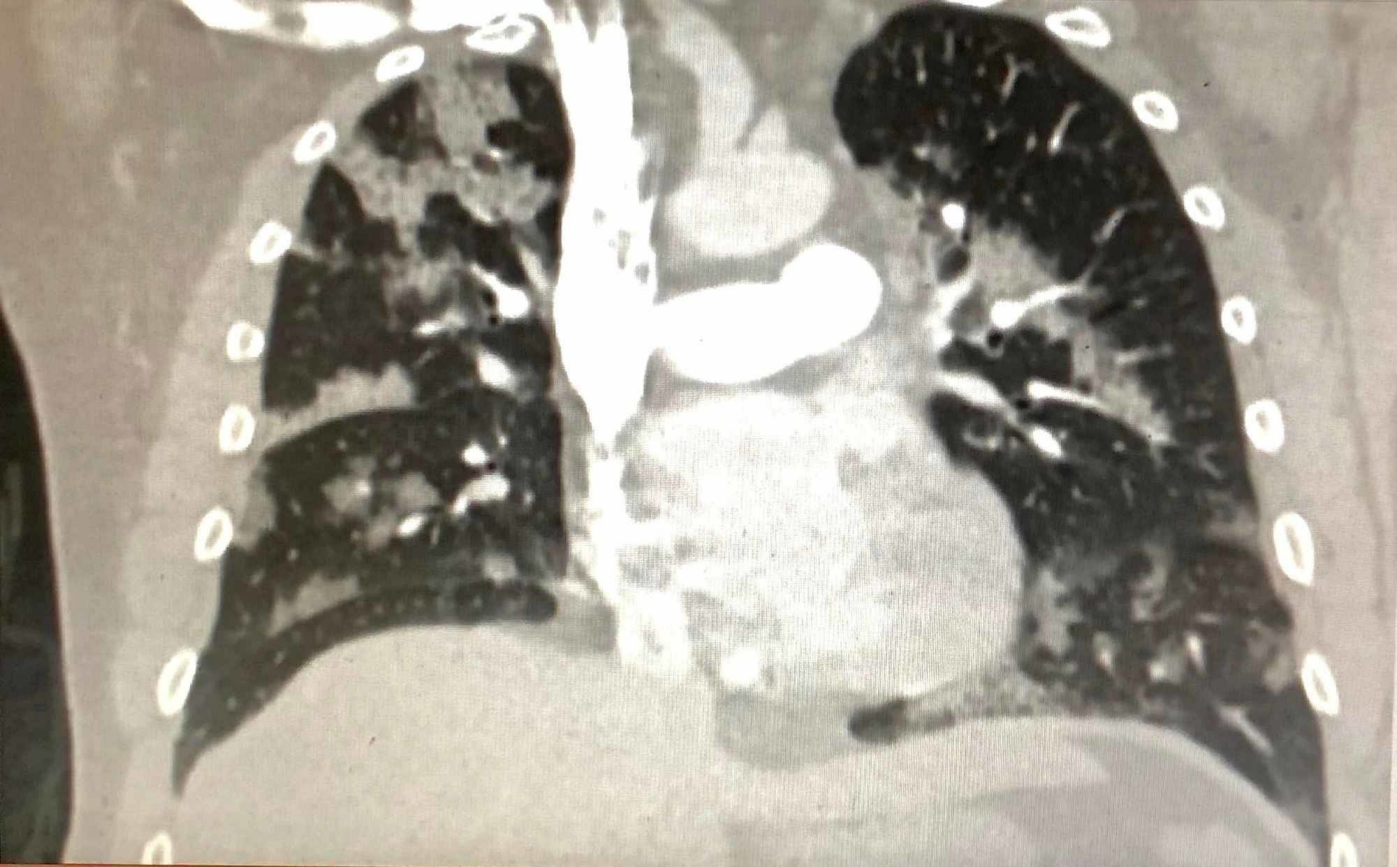
Computed tomography (CT) of the chest (coronal view) showing pulmonary edema

In the ER, he was noted to have multiple electrocardiogram (EKG) abnormalities and hypoxemia. Due to concern for cardiac etiology of his symptoms, he was flown via helicopter to a referral center ER. His EKG showed left atrial enlargement and a hyperdynamic QRS wave without evidence of acute myocardial infarction (Figure 1). He continued to experience shortness of breath and hypoxemia to 88% O_2_ on room air, and became tachypneic. He responded well to 4L nasal cannula with O_2_ saturation on pulse oximetry of 96%. Additionally, vital signs were a temperature of 98.8, blood pressure of 155/85, heart rate of 88, and respiratory rate of 22 that resolved to 16 with initial therapy. Computed tomography (CT) of the chest revealed pulmonary edema without evidence of pulmonary embolism (Figure 2). Lab results revealed a troponin I of 0.29 ng/mL, a brain natriuretic peptide (BNP) of 199 pg/mL, and leukocytosis of 23.3 K/uL. Otherwise, his complete metabolic panel, urine drug screen, and lactate were unremarkable.

**Figure 3:**
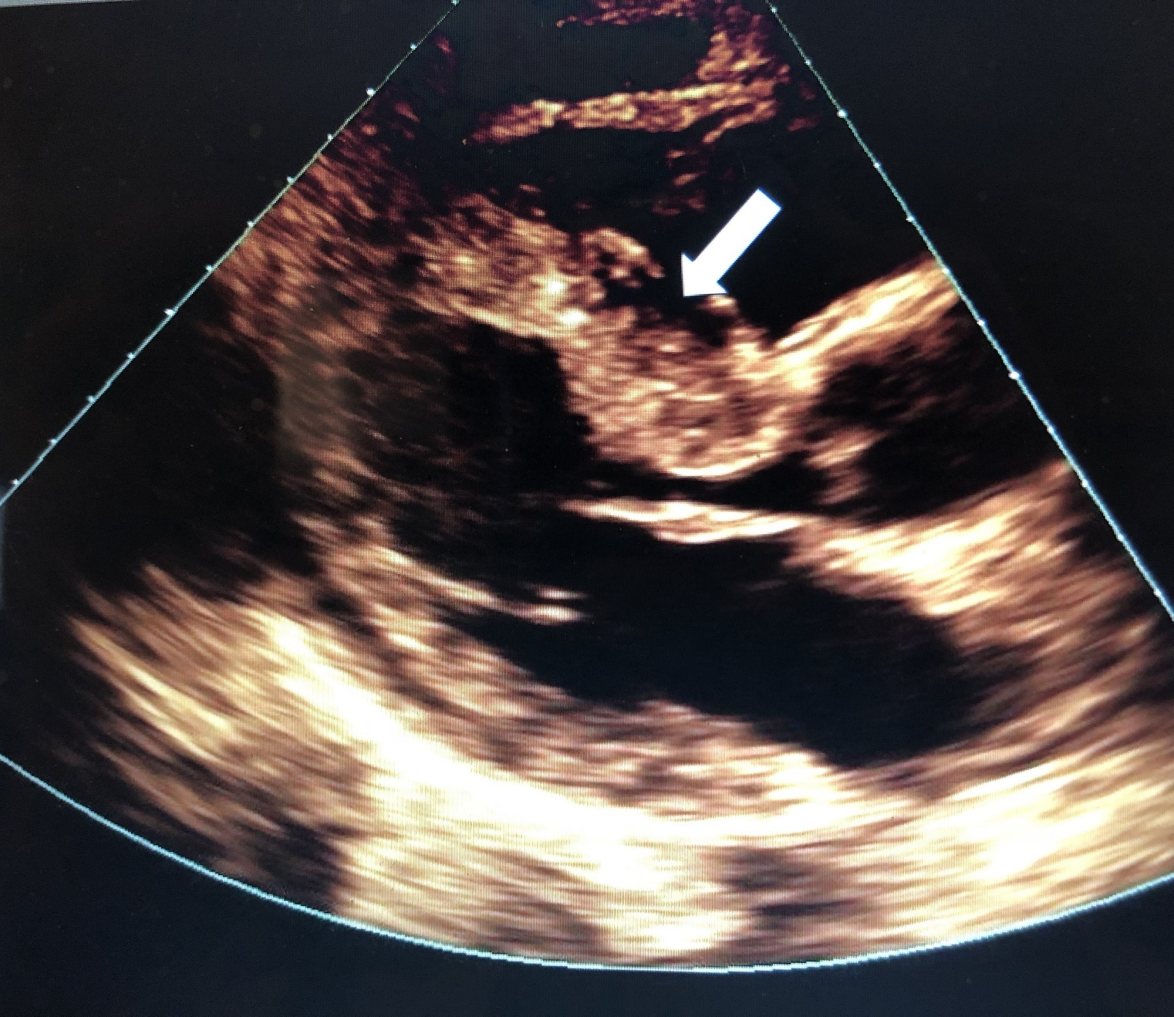
Parasternal long axis with arrow pointing to hypertrophic septum

**Figure 4:**
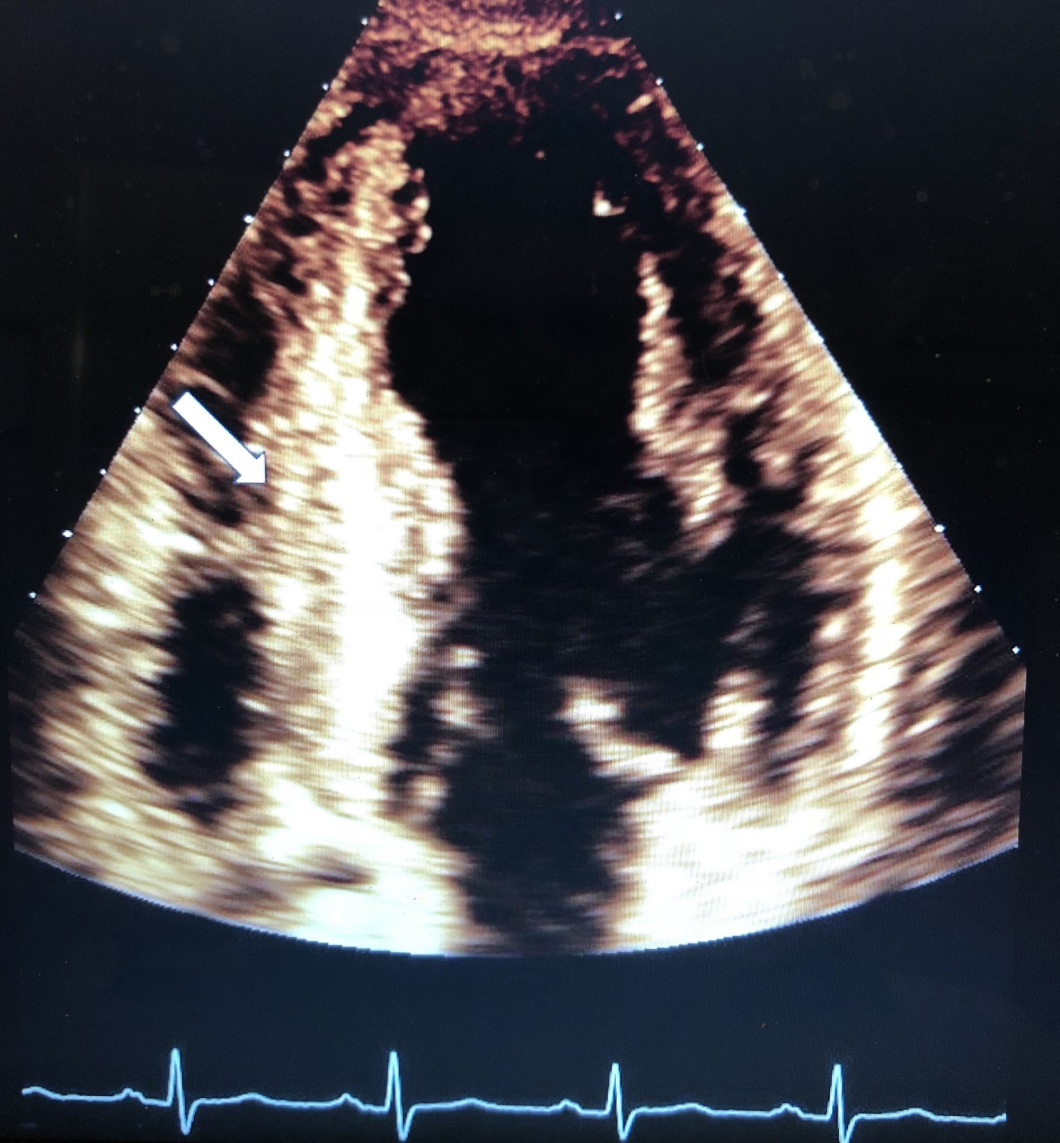
Apical four-chamber view with arrow pointing to hypertrophied septum

**Figure 5:**
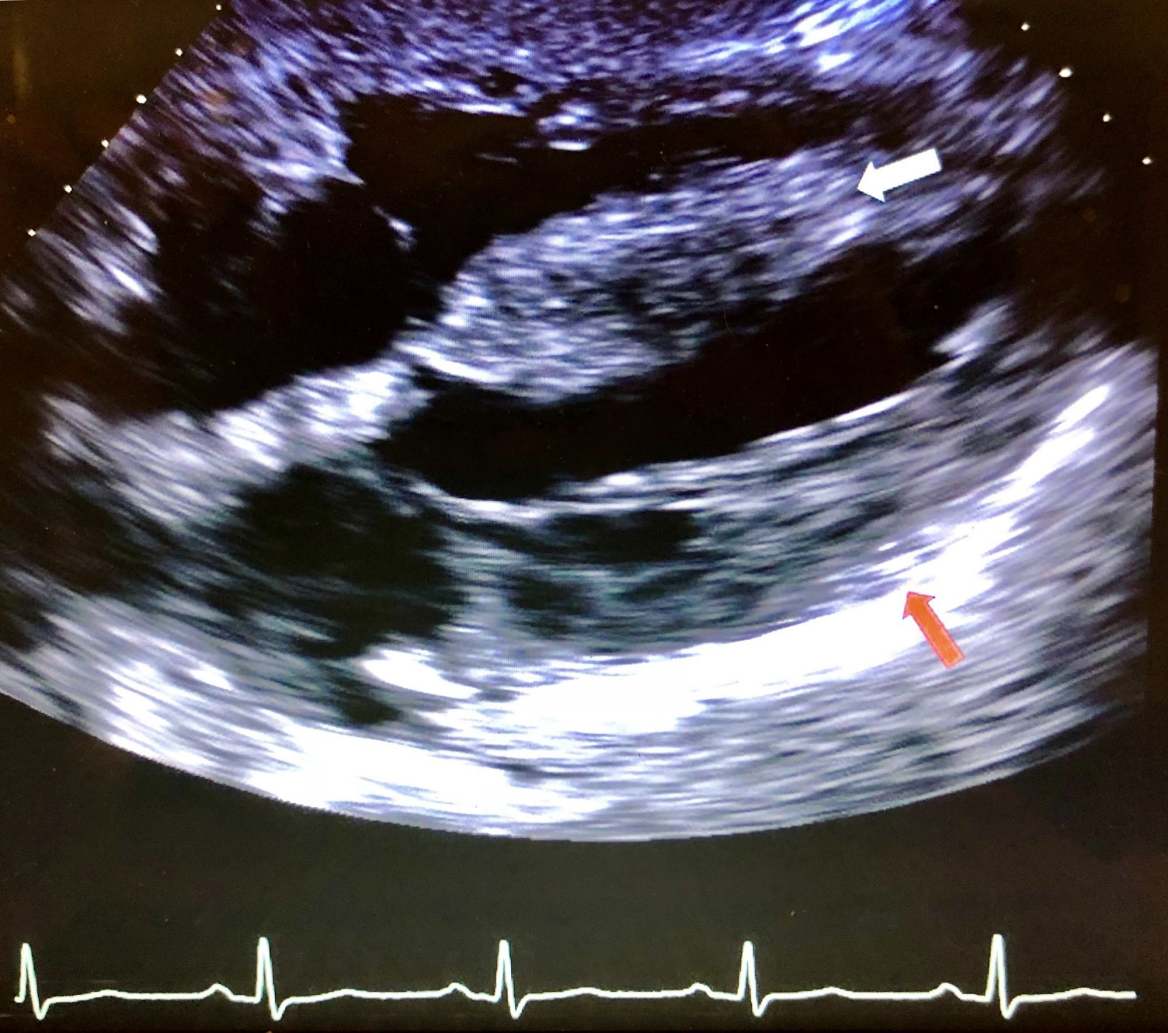
Subxiphoid window with white arrow pointing to hypertrophied septum and red arrow pointing to hypertrophied left ventricular wall

During the remainder of his ER course, he did not require further respiratory intervention. He was given a dose of 40 mg intravenous (IV) Lasix for pulmonary edema and gradually improved as a result. He was admitted to the inpatient unit where an echocardiogram revealed severe asymmetric septal-hypertrophy and hypertrophic obstructive physiology with left ventricular outflow tract (LVOT) resting pressure gradient of 31 mmHg, mild left atrial-chamber enlargement, and moderate mitral valve regurgitation, but no left ventricle wall motion abnormality (Figures 3-5). Percutaneous coronary angiography revealed mild-diffuse luminal irregularities of the left main coronary artery, first diagonal branch, third obtuse marginal branch, and right coronary artery (which was dominant).

He was placed on long-acting diltiazem and amlodipine was discontinued. Additionally, he was started on low dose aspirin with dietary risk factor modification, daily atorvastatin, good oral hydration, and a low sodium diet. Cardiology follow up was established and the remainder of his hospital course was uncomplicated with a continued resolution of presenting symptoms.

## Discussion

We are taught that hypertrophic obstructive cardiomyopathy (HOCM) is present in young athletes. The initial presentation of HOCM in an athlete over the age of 50 makes this case unique. When a person over the age of 50 presents to the ER with acute shortness of breath and elevated troponin, previously undiagnosed decompensated HOCM is very low on the differential, if it is even considered.

The first important learning point, in this case, is to identify the cognitive bias associated with the idea that HOCM is a disease of adolescents. This bias had been propagated through our training as the majority of HOCM diagnosis are made in young athletes. Though this case is relatively rare, we must recognize that HOCM is not a disease exclusively confined to adolescents, given the estimated genetic preponderance of 1 in 500 people having some form of this disease. The burden of previously undiagnosed individuals with subclinical disease is likely encountered much more than providers anticipate in primary care and emergency medicine.

When we think of the initial presentation of HOCM, our interest is piqued when a patient describes a syncopal event during exercise. However, that is the textbook presentation of an undiagnosed adolescent. In this case, the patient’s initial presentation was dyspnea due to pulmonary edema and elevated troponin. This closely mimicked the presentation of acute decompensated congestive heart failure (CHF). Except, the patient’s blood pressure was not grossly elevated, it was 155 systolic/82 diastolic and his BNP was only 199, which is not consistent with severe CHF.

People with HOCM do suffer from varying degrees of coronary microvascular dysfunction, given the myocardial fiber disarray inherent in this disease [7-8]. Superimposed atherosclerotic coronary artery disease (CAD) further limits myocardial perfusion capabilities in these at-risk hearts [9]. Therefore, mild diffuse CAD that would otherwise be asymptomatic in a normal heart may precipitate acute decompensation in an older athlete with HOCM.

## Conclusions

In conclusion, the burden of HOCM in the population is larger than most providers see in practice with many cases likely being subclinical. Lacking a previous diagnosis of HOCM does not exclude an adult athlete from having this disease since a first decompensation event can have a temporal pattern not regularly taught in training, though this is uncommon. A first decompensation event in an adult athlete may have a clinical presentation that mimics other acute chest pathophysiology and may not fit the text-book presentation of this disease, though further research will be necessary to elucidate this.
